# The complete chloroplast genome sequence of a Chinese endemic plant *Dendrobium hancockii* Rolfe (Orchidaceae)

**DOI:** 10.1080/23802359.2019.1687036

**Published:** 2019-11-08

**Authors:** Zhenyu Hou, Yu Jiang, Chao Li, Zhitao Niu, Xiaoyu Ding

**Affiliations:** a College of Life Sciences, Nanjing Normal University, Nanjing, China;; b Jiangsu Provincial Engineering Research Center for Technical Industrialization for Dendrobium, Nanjing Normal Uniwersity, Nanjing, China

**Keywords:** *Dendrobium hancockii*, chloroplast genome, phylogeny, sect. *Dendrobium*

## Abstract

*Dendrobium hancockii* Rolfe is a rare and endangered species endemic to China, with great medicinal value. Here, the first complete chloroplast genome sequence of *D*. *hancockii* was reported and characterized. The cpDNA exhibited the typical quadripartite structure of four parts: a long single-copy region, a short single-copy region and two inverted repeats. It encodes 106 genes, consisting of 72 unique protein-coding genes, 30 unique tRNA gene, and 4 unique rRNA genes. The phylogenetic analysis indicated that *D*. *hancockii* is basal-most species for the sect. *Dendrobium*.

*Dendrobium* is one of the largest genera of Orchidaceae, with about 80 species distributed in China. Although it is composed of several sections, the species in sect. *Dendrobium* are favored for their high horticultural and medicinal value. For example, *Dendrobium hancockii* Rolfe, the basal-most species in this sect., contains great medicinal value. It is a rare and endangered species endemic to China, mainly distributes in the Gansu, Shanxi, Hubei, Guangxi, Sichuan, Guizhou, and Yunnan provinces. It grows on forest trunks and valley rocks of 700–1500 m above sea level. Here, to verify its phylogenetic location and provide more genomic resources useful for promoting its identification, utilization and conservation, we report and characterize the complete chloroplast genome (cpDNA) of *D*. *hancockii*.

The plant species of *D. hancockii* was sampled from Yunnan province of China (98.48E, 25.01N, voucher specimen: JiangTC6), and grow in the green house in Nanjing Normal University, Jiangsu, China. The fresh leaves of *D. hancockii* were harvested for the DNA extraction. Total genomic DNA was isolated using a modified CTAB method (Doyle and Doyle 1987). The DNA sample that met the quality requirement (concentration >50 ng/μl, A260/A280 = 1.8–2.0, and A260/A230 > 1.8) were prepared for sequencing. Approximately 5.0 Gb of 150 bp pair-end reads were yielded using an Illumina Hiseq4000 sequencer. The raw reads were trimmed and assembled into contigs with the guidance of SOAP de novo (v2.04) and CLC Genomics Workbench 8.0 adapted from Niu et al. 2017. Gaps and junctions between inverted repeat (IR) regions and single copy (SC) regions were verified by PCR amplification. Gene annotation was performed via DOGMA (Wyman et al. 2004) and tRNAscan-SE 1.21 (Schattner et al. 2005). The completed sequence of chloroplast genomes was deposited in DDBJ (Accession no. LC_500592).

The newly sequenced chloroplast genome of *D*. *hancockii* is 151,688 bp in length and exhibited the typical quadripartite structure, containing a pair of inverted repeats (IRs) of 26,295 bp separated by a large single-copy (LSC) region of 84,707 bp, and a small single-copy (SSC) region of 14,391 bp. The genome consisted of a total 106 genes, including 72 protein-coding genes, 30 tRNA genes, and 4 rRNA genes. The GC content accounted for 37.56% of whole genome sequence, which is similar to the results of other related *Dendrobium* species. The corresponding GC contents of the LSC, SSC, and IR region were 35.10%, 30.89%, and 43.35%, respectively.

To determine the phylogenetic position of *D*. *hancockii*, phylogenetic analysis was conducted using 9 reported representative *Dendrobium* species with *Phalaenopsis aphrodite* (NC_007499) and *Phalaenopsis equestris* (JF_719062) as outgroups. The sequences were aligned using MAFFT v. 7.427 (Nakamura et al. 2018). Using the GTRGAMMA substitution model and employing RAxML 8.0.2 with 100 bootstrap replicates (Stamatakis 2014), we reconstructed a maximum-likelihood (ML) tree and found *D*. *hancockii* is a separate branch without species complex (Figure 1). These results were similar to that exhibited in the previous studies based on cpDNA fragments, which indicated that *D*. *hancockii* is located at the base of the sect. *Dendrobium* (Xiang et al. 2013).

**Figure 1.**
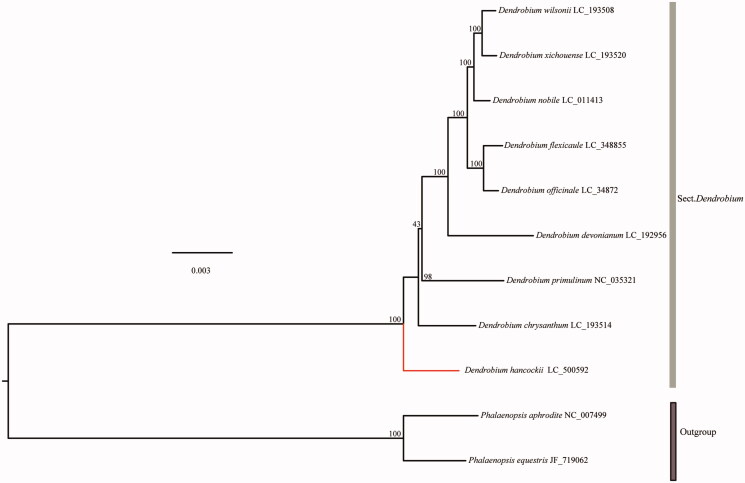
Maximum-likelihood tree of *Dendrobium* species based on the whole chloroplast genome sequences with *Phalaenopsis aphrodite* and *Phalaenopsis equestris* as outgroups. Numbers near the nodes represent ML bootstrap values.
